# Monozygotic Twins Concordant for Common Variable Immunodeficiency: Strikingly Similar Clinical and Immune Profile Associated With a Polygenic Burden

**DOI:** 10.3389/fimmu.2019.02503

**Published:** 2019-11-22

**Authors:** Susana L. Silva, Mariana Fonseca, Marcelo L. M. Pereira, Sara P. Silva, Rita R. Barbosa, Ana Serra-Caetano, Elena Blanco, Pedro Rosmaninho, Martin Pérez-Andrés, Ana Berta Sousa, Alexandre A. S. F. Raposo, Margarida Gama-Carvalho, Rui M. M. Victorino, Lennart Hammarstrom, Ana E. Sousa

**Affiliations:** ^1^Faculdade de Medicina, Instituto de Medicina Molecular João Lobo Antunes, Universidade de Lisboa, Lisbon, Portugal; ^2^Centro de Imunodeficiências Primárias, Centro Académico de Medicina de Lisboa, Centro Hospitalar Universitário Lisboa Norte and Faculdade de Medicina da Universidade de Lisboa and Instituto de Medicina Molecular, Lisbon, Portugal; ^3^Centro Hospitalar Universitário Lisboa Norte, Hospital de Santa Maria, Lisbon, Portugal; ^4^Faculty of Sciences, BioISI-Biosystems & Integrative Sciences Institute, University of Lisboa, Lisbon, Portugal; ^5^Department of Medicine, Cancer Research Centre (IBMCC, USAL-CSIC), Cytometry Service (NUCLEUS), Institute of Biomedical Research of Salamanca (IBSAL), University of Salamanca (USAL), Salamanca, Spain; ^6^Biomedical Research Networking Centre on Cancer-CIBER-CIBERONC, Number CB16/12/00400, Institute of Health Carlos III, Madrid, Spain; ^7^Department of Laboratory Medicine, Karolinska Institutet, Stockholm, Sweden

**Keywords:** CVID, flow-cytometry, polygenic disease, genetics, WES, monozygotic twins

## Abstract

Monozygotic twins provide a unique opportunity to better understand complex genetic diseases and the relative contribution of heritable factors in shaping the immune system throughout life. Common Variable Immunodeficiency Disorders (CVID) are primary antibody defects displaying wide phenotypic and genetic heterogeneity, with monogenic transmission accounting for only a minority of the cases. Here, we report a pair of monozygotic twins concordant for CVID without a family history of primary immunodeficiency. They featured a remarkably similar profile of clinical manifestations and immunological alterations at diagnosis (established at age 37) and along the subsequent 15 years of follow-up. Interestingly, whole-exome sequencing failed to identify a monogenic cause for CVID, but unraveled a combination of heterozygous variants, with a predicted deleterious impact. These variants were found in genes involved in relevant immunological pathways, such as *JUN, PTPRC, TLR1, ICAM1*, and *JAK3*. The potential for combinatorial effects translating into the observed disease phenotype is inferred from their roles in immune pathways, namely in T and B cell activation. The combination of these genetic variants is also likely to impose a significant constraint on environmental influences, resulting in a similar immunological phenotype in both twins, despite exposure to different living conditions. Overall, these cases stress the importance of integrating NGS data with clinical and immunological phenotypes at the single-cell level, as provided by multi-dimensional flow-cytometry, in order to understand the complex genetic landscape underlying the vast majority of patients with CVID, as well as those with other immunodeficiencies.

## Background

Monozygotic twins provide a unique opportunity to evaluate the relative contribution of genome and environment to the development and evolution of the immune system throughout life (1–3). A recent study on a large twin cohort suggests that life experience is the main determinant, challenging the importance of genetic background (2). Monozygotic twins (MZ) also provide a valuable tool to investigate complex diseases of the immune system like Common Variable Immunodeficiency (CVID).

CVID is defined overall by a marked decrease in serum IgG and IgA, normal or decreased serum IgM, poor antibody responses to vaccines and absence of other identifiable causes for hypogammaglobulinemia (4). However, almost all components of the immune system may feature alterations, with high heterogeneity between patients (5, 6). Given the marked clinical heterogeneity, multiparametric flow-cytometry is instrumental to detail the individual immune profile (7–10), allowing personalized approaches to treatment and monitoring of co-morbidities (7, 10).

A genetic basis for CVID was already recognized in 1968 (11), though the identification of the underlying molecular defects has been hampered in the majority of patients, which usually do not have a family history. In recent years, next generation sequencing (NGS) strategies, including genome-wide association and whole-exome/genome sequencing studies (WES/WGS) (9, 12–19), have facilitated the identification of an increasing list of genes, with heterozygous or biallelic variants associated with monogenic CVID (9, 12–20). Nevertheless, monogenic transmission is currently assumed in only 15–25% of the patients with CVID, in association with pathogenic variants in genes related mostly to B-cell activation, T-cell signaling and cytokine expression (14, 15, 17, 20). Conversely, CVID is likely to be polygenic in the majority of patients, resulting from multiple epistatic interactions with cumulative effects (12, 21). Consistently, CVID may develop clinically at any age, suggesting progressive and cumulative deterioration of B-cell functions, in a putative multifactorial pathogenic process (8, 22).

The contribution of non-heritable influences, such as infectious exposure, to the establishment of the clinical and immunological phenotypes in CVID is also recognized (23). The possible role of viral infections as triggers to disease onset, and the impact of microbiome are illustrative ongoing debates (23, 24). Twin studies thus provide a powerful model to dissect the relative contributions of heritable and non-heritable variables to the establishment of clinical and immunological profiles, but have so far been poorly explored, given the rarity of these cases (2, 3).

We provide here the first report of MZ twins concordant for CVID. Extensive phenotypic profile of circulating B and T-cell subsets was obtained by flow-cytometry at diagnosis and during 15-years follow-up. The twins featured a remarkably similar immunologic and clinical profile, despite the absence of a recognizable monogenic cause, being 50 years-old, and having lived apart for many years. Importantly, WES allowed us to identify a combination of variants with putative impact in the immune system that is not shared by their progenitors or progeny, supporting a polygenic basis for the CVID phenotype and their concordant immune evolution.

## Case Presentation

CVID diagnosis was performed at 37 years of age in a pair of Caucasian MZ twins, living in Lisbon, Portugal, born from non-consanguineous parents and with no family history of primary immunodeficiency. They presented a remarkably similar clinical and immunological profile throughout 15 years of follow-up, despite distinct living conditions (jobs, housing, and nuclear families) since age 25.

**Figure 1:**
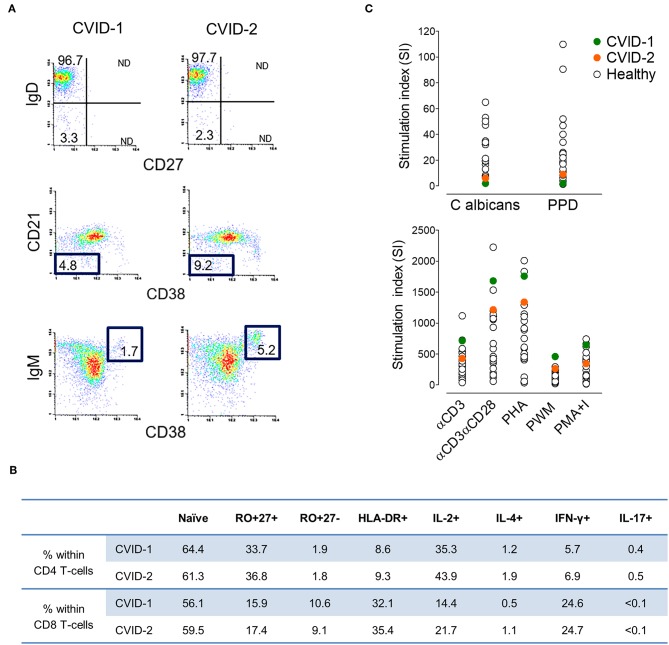
Immune phenotype at CVID diagnosis at age 37 in the MZ twins. **(A)** Representative plots of the flow-cytometry analysis of switched-memory B cells (top), CD21^low^CD38^low^ B cells (middle) and transitional B cells (bottom). Numbers represent the percentage of the given population within CD19^+^ cells (3.0%/4.2% in case-1/case-2, respectively). **(B)** Frequency of naïve and memory subpopulations, expression of activation marker HLA-DR^+^, and frequency of cells producing IL-2, IL-4, IFN-γ, and IL-17 within CD4 and CD8 T cells (1,777/1,380 lymphocytes/μL; CD4 T cells 42.2%/43.6%; CD8 T cells 44.1%/39.8%; in cases 1/2, respectively) **(C)** Lymphoproliferative responses upon culture with antigens (top) and mitogens (bottom) in comparison with healthy adult individuals.

At diagnosis, both twins featured severe hypogammaglobulinemia, with very low serum IgG (<0.3 and 1.65 g/L, cases 1 and 2, respectively), undetectable IgA (<0.25 g/L) and IgM (<0.19 g/L), and no increase in titres of specific IgG upon polysaccharide pneumococcal and conjugated *Haemophilus influenzae* vaccinations. They had a low frequency of circulating total and memory B cells, particularly of switched-memory B cells, with no expansions of transitional B cells or of cells expressing low levels of CD21 (Figure 1A), thus featuring an identical EuroClass classification B^+^smB^−^Tr^norm^21^norm^ (25). They also exhibited comparable naïve/memory subset distribution and expression of activation markers in CD4 and CD8 T cells (Figure 1B), with a defect in CD45 alternative splicing leading to the persistence of CD45RA in memory/effector T cells. Functional studies in T cells revealed an equal production of IL-2, IL-4, IFNγ, and IL-17 (Figure 1B) and impairment of proliferative responses to recall antigens, despite the relative high levels of proliferative responses to mitogens (Figure 1C).

**Figure 2:**
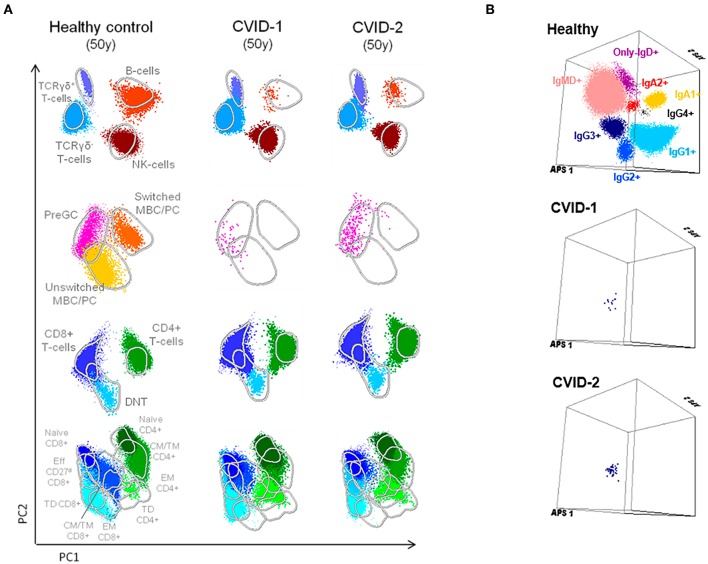
Supervised flow-cytometric analysis of blood lymphocytes in MZ twins concordant for CVID at age 50. **(A)** Principal component analysis (PCA) multidimensional view of the distribution of major lymphocyte subsets analyzed with the EuroFlow PID orientation tube in 1 × 10^6^ peripheral blood leukocytes. **(B)** Distribution of memory B cells according to the surface membrane expression of the IgH-isotypes (IgM, IgD, IgG1, IgG2, IgG3, IgG4, IgA1, and IgA2) in 5 × 10^6^ peripheral blood leukocytes analyzed from an age-matched healthy donor and the two twins with CVID (7, 8, 26).

Along the follow-up there was concordant evolution of their immunological profile, shown by the PCAs obtained at age 50 with the EuroFlow protocols (Figure 2A). The detailed phenotypic evaluation of circulating B cells revealed severe B-cell depletion (total B cells <1%) in both, with residual preservation of IgG3^+^CD27^−^ memory B cells detected by high-sensitivity methods (7, 26, 27) (Figure 2B). This profile, which reflects extreme deterioration of IgG-switching capacity in memory B cells, is compatible with the most severe (CVID-6) subgroup, as recently reported in the literature (7).

The type and severity of clinical manifestations before diagnosis and during follow-up have also been very similar. Both twins featured upper and lower respiratory infections since their twenties, with progressively increased frequency leading to several hospital admissions for pneumonia; bilateral bronchiectasis; and nasal polypectomy at age 32 in both. Upon diagnosis, intravenous IgG replacement was initiated, leading to serum IgG levels above 800–900 mg/dL and a marked decline in the frequency of infectious episodes. Both maintained persistent sinusitis, with recurrent exacerbations mostly in conjunction with *H. influenza* infection, and intermittent non-infectious diarrhea compatible with minor chronic lymphocytic infiltration, observed in duodenal and colon mucosa.

**Figure 3:**
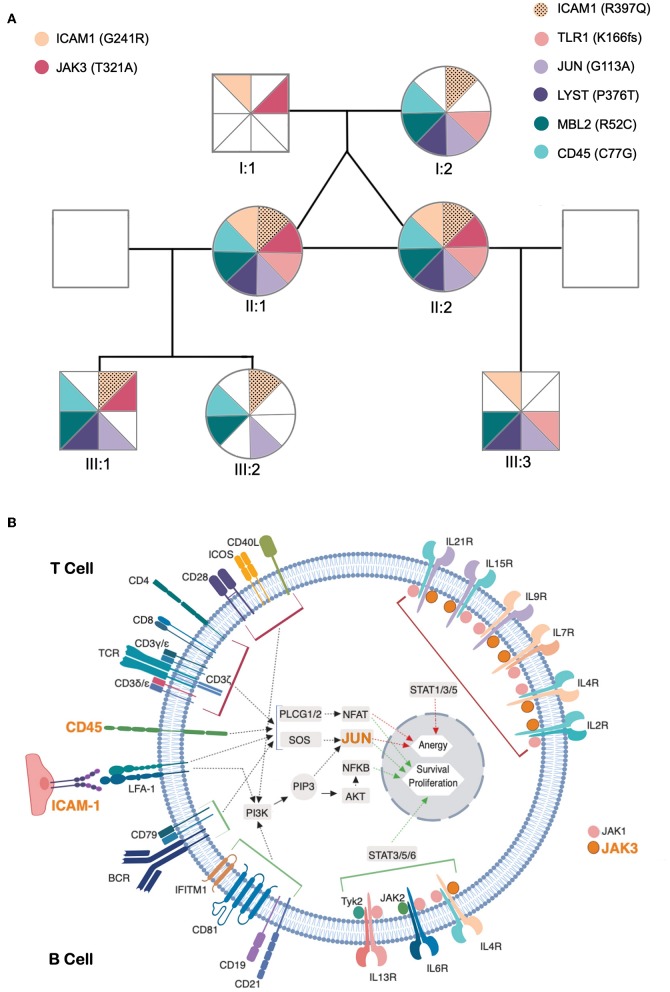
Identified genetic variants and predicted functional impact on B and T-cell activation pathways. **(A)** Family tree of the patients. The sectors and color patterns represent the 8 genetic variants identified in the 7 listed genes, and their presence/absence in both the progenitors and the children of the two siblings. Each sector corresponds to one specific variant. Paternally inherited variants are represented on the left, and maternal variants on the right, with a color code per gene, next to the gene ID and identified variant (in parenthesis). The black dotted pattern highlights the maternal variant in the case of *ICAM*, for which the patients have two altered alleles. **(B)** Role of affected genes in T and B-cell activation processes. The figure represents a generic lymphoid cell with the major membrane receptors, signaling molecules and transcription factors (identified by their names) and intracellular signaling pathways (identified by arrows) involved in the activation process. Red and green arrows represent the final stages of the lymphocyte activation pathways leading to the expression of genes that promote anergy (red) or survival/proliferative responses (green) within the cell nucleus. The top half of cell highlights proteins and processes specific for T cell activation and the bottom half those specific for B-cell activation. The names of proteins encoded by genes that present potentially pathogenic variants are presented in orange. The figure compiles all the information that was retrievable from pathways databases and literature search regarding the connections between genes presented in **(A)** and the B and T cell activation pathways. Of these 7 genes, *LYST* did not present any connection to these processes, whereas two others (*MBL2* and *TLR1*) have reported functions in lymphocyte activation but their connection to signaling pathways remains unclear and are thus not represented in the figure.

This remarkably similar profile in MZ twins strongly suggested that the genetic background is the main contributor to their clinical and immunological evolution. We therefore sequenced their whole exome (WES) using DNA extracted from blood samples. We focused the analysis on genes involved in immunological pathways, identifying SNVs with a non-synonymous coding effect predicted to be either probably damaging or deleterious (28, 29), or frame shift variants (see Methods). We also looked for variants affecting mRNA splice sites, but we found no variants in genes reported as monogenic causes of CVID. In fact, WES analysis was unable to reveal a monogenic cause for CVID. However, we identified in both a combination of 7 heterozygous SNVs, occurring in *JAK3, JUN, LYST, MBL2, ICAM1, and TLR1* (Table 1 and Figure 3A). None of these 6 genes has previously been shown to be associated with *monogenic* CVID (15, 32). A compound heterozygous change, combining one variant from each parent, was found in *ICAM1*.

**Table 1:** Selected SNVs with impact in the immune system identified by WES.

**Gene**	**Change coordinates**	**Ensembl transcript ID**	**Protein variant (HGVS)**	**dbSNP ID**	**ExAC (MAF EUR)**	**PolyPhen**	**SIFT**	**Immunological role of the gene-encoded protein**
*JAK3*	chr19:17952472 T/C	ENST00000428406 ENST00000458235 ENST00000527670 ENST00000534444	T321A	NA	NA	PD	T	Member of Janus kinase family of tyrosine kinases; Cytokine receptor-mediated intracellular signal transduction
*ICAM1*	chr19:10394792 G/A	ENST00000264832	G241R	rs1799969	0,1102	PD	D	Cell surface glycoprotein with major role in cell-cell adhesion, in endothelia and immune cells
	chr19:10395468 G/A	ENST00000423829 ENST00000264832	R397Q	rs5497	0,0006	PD	D	
*JUN*	chr1:59248405 C/G	ENST00000371222	G113A	rs1462279538	NA	PD	D	Transcription Factor AP-1 interacts with specific target DNA sequences to regulate gene expression in the immune system
*LYST*	chr1:235972992 G/T	ENST00000536965 ENST00000389794 ENST00000389793	P376T	rs770362521	3 ×10^−5^	PD	T	Regulates intracellular protein trafficking in endosomes; Mutations associated with Chediak-Higashi syndrome with impaired cytotoxic lymphocyte function (30)
*TLR1*	chr4: 38799956; NM_003263.4:c.497del	ENST00000308979 ENST00000502213	K166fs	rs761749628	0,0003454	NA	D	Member of the Toll-like receptor family, with a role in pathogen recognition and activation of innate immunity. Identified as a critical mediator of intestinal immunity
*MBL2*	chr10:54531242 G/A	ENST00000373968	R52C	rs5030737	0,076	PD	D	Belongs to collectin family. Important element in the innate immune system Variants associated with susceptibility to autoimmunity and infections (31)
*PTPRC*	chr1:198665917 C/G	ENST00000352140 ENST00000367376 ENST00000418674 ENST00000442510 ENST00000529828	C77G	rs17612648	0,016	S	N/A	Important for efficient T and B-cell antigen receptor signal transduction CD45RA persistence in memory T cells in alternative splicing defect

*HGVS, human genome variation society; NA, not available; MAF, minor allele frequency; EUR, European; PD, probably damaging; S, synonimous; T, tolerated; D, deleterious*.

In face of the defect in CD45 alternative splicing observed in T cells, we additionally looked at variants in *PTPRC (CD45)*. We found the twins were heterozygous carriers of C77G (Table 1 and Figure 3A), a heterozygous variant that was previously reported in heterozigosity in CVID patients (33, 34).

In order to elucidate the segregation of the identified variants and their possible associations with clinical manifestations and/or immunological profile, their parents and children were also evaluated. Neither the progenitors nor the progeny had any severe/recurrent infections or immune-mediated diseases, and all had normal levels of serum IgG, IgA, and IgM, except for one son with a past-history of autoimmune thrombocytopenia (III:3, Figure 3A). Parents and children were genotyped for the 8 genetic variants (Figure 3A) and the variants considered were found to be split between parents, and none of the descendants inherited the combination of variants observed in the twins. Due to the frequent onset of CVID-associated clinical manifestations in adulthood, prospective follow-up of the offspring, particularly of the son with a past-history of autoimmune thrombocytopenia, will be important to complete our current interpretation of NGS data.

## Methods

### Patients and Relatives

Longitudinal data obtained from a pair of MZ twins followed at Centro de Imunodeficiências Primárias, of Lisbon Academic Medical Center. The patients met the European Society for Immunodeficiencies (ESID) diagnostic criteria for CVID at the time of enrolment (35). Patients and relatives gave written informed consent. The study was approved by the ethical boards of the Faculty of Medicine of University of Lisbon and Centro Hospitalar Universitário Lisboa Norte.

### Immunological Studies

Phenotypic analysis was performed by flow-cytometry during follow-up using previously described protocols (8, 36, 37) and different cytometers (FACSCalibur, FACSCanto and LSR-Fortessa X-20 flow-cytometers, Becton/Dickinson Biosciences (BD), San José, CA). Briefly, stainings with monoclonal antibodies were performed in whole blood after red blood cells lysis using BD FACS Lysing Solution (BD Biosciences), and a minimum of 100,000 lymphocytes were acquired per sample with data analyzed using CellQuest Software (BD Biosciences) and FlowJo Software (Tree Star Inc., Ashland, OR). More recently, 10^7^ nucleated cells were stained with the EuroFlow 12-color Ig-isotype B-cell tube and bulk-lyse standard operating procedure (SOP; www.EuroFlow.org), ≥5 × 10^6^ leukocytes were acquired in LSR-Fortessa X-20, with instrument set-up and calibration performed according to the EuroFlow SOP (38), and data analyzed with Infinicyt software (Cytognos S.L., Salamanca, Spain).

Cytokine production was assessed at the single-cell level in peripheral blood mononuclear cells (PBMC) freshly isolated by Ficoll-Hypaque density gradient (Amersham Pharmacia Biotech, Uppsala, Sweden), as described (39). Briefly, after a 4-h culture with phorbol myristate acetate (PMA; 50 ng/mL, Sigma-Aldrich) plus ionomycin (500 ng/mL; Calbiochem, Merck Biosciences, Nottingham, U.K.), in the presence of brefeldin A (10 μg/mL; Sigma-Aldrich), PBMC were surface stained, fixed, permeabilized, and stained intracellularly with monoclonal antibodies against IL-2, IL-4, IFN-γ, and IL-17, as described (36). Flow-cytometric analysis was subsequently performed as described above.

Lymphocyte proliferation was evaluated as follows: 10^5^ freshly isolated PBMCs were cultured in a 96-well plate in the absence or presence of mitogens PHA (20 μg/mL), anti-CD3 (1 μg/mL), anti-CD3 anti-CD28 (1 μg/mL), PWM (1 μg/mL) and PMA (50 ng/mL)+ ionomycin (500 ng/mL) and antigens *Candida albicans* (40 μg/mL) and PPD (5 μg/mL), for 3 and 6 days, respectively, at 37°C, 5% CO_2_. Proliferation was assessed by 3H-Thy (Amersham Pharmacia Biotech) incorporation in the last 8 h of culture. Results were expressed as stimulation indexes (SI) which represents the ratio of the mean counts per minute (cpm) in the presence of a given mitogen or antigen over the mean cpm in the absence of the stimulus.

### Whole Exome Sequencing

Genomic DNA was extracted from peripheral blood, subjected to library construction using the Agilent Sure Select Human All Exon 50 Mb kit (Agilent Technologies) and sequenced on a Hiseq2000 Illumina sequencer (BGI-Shenzhen, China) (40). Low-coverage and low-quality Single-Nucleotide Variants (SNVs) were removed as described (41). High-quality reads were aligned to the reference human genome (GRCh37/hg 19) and annotated with SnpEff Tool. Non-synonymous SNVs predicted to be probably damaging or deleterious [either by PolyPhen 2.2.2 (28) or SIFT 5.1.1 (29)], or frameshift variants, regardless of the minor allele frequency were filtered and prioritized. Criteria to further narrow down the candidate gene list were applied as previously described (42). Extensive search was performed in the literature, interactome and pathways databases regarding the roles of identified genes in the immune system, particularly concerning their involvement in B and T-cell activation pathways, illustrated in Figure 3B.

## Discussion

Here we report the first study of a pair of MZ twins concordant for CVID. They featured remarkable similarity in clinical and immunological phenotypes at diagnosis and during 15-year follow-up, and the analysis of WES data did not identify pathogenic variants in genes previously reported in association with monogenic CVID. In contrast, we identified 7 non-synonymous coding variants with predicted damaging/deleterious impact on the 6 proteins coded by the involved genes. These genes integrate relevant immune pathways and are therefore likely to have a clinical impact, supporting a polygenic burden for CVID, as well as to constrain the evolution of immunological profiles, leading to the high degree of similarity observed between the twins, despite having led separate lives for over 25 years. The clinical and immunological outcome in the twins likely results from the accumulation of distinct functional impairments, due to variants in genes related to critical immunological pathways (12, 21), as none of the identified heterozygotic variants can independently explain their clinical/immunological picture. Consistent with this polygenic model, there is a potential combinatorial impact of the observed SNVs in B and T-cell activation, as illustrated in Figure 3B. The absence of clinical and immunological manifestations in close family members also argues in favor of a pathogenic burden derived from the unique combination of variants shared by the twins.

There are previous reports of CVID patients who are heterozygous carriers of C77G in *CD45* (33, 34). CD45 is known to be important for efficient T and B-cell antigen receptor signal transduction and for control of signaling thresholds through TCR (31, 43). C77G is the most common cause of *CD45* abnormal splicing in European populations (44), which leads to the persistence of CD45RA in memory T cells (45, 46). The twins are heterozygous for C77G and exhibit a strong mitogenic response to anti-CD3, but markedly diminished proliferative responses to the tested recall antigens. Our data add to previous reports (33, 34) favoring a role for this SNV in a polygenic scenario for CVID, although the frequency of (heterozygous) carriers of C77G in *CD45* was not increased in a large CVID cohort (33) and different clinical phenotypes have been associated with C77G in *CD45*.

Obviously, we cannot exclude a role for other variants, such as non-coding variants, in the genes that we mentioned, nor in other genes, that may impact in the clinical and immunological phenotype of the twins. Nevertheless, it is worth noting that 4 out of the 7 selected genes with variants act on both T and B-cell receptor signaling-pathways, namely *JUN, CD45* (*PTPRC*), and *ICAM1* (Figure 3B); and *MBL2* and *TLR1*, influence these processes through as yet unidentified mechanisms (see below) (30, 31, 46–53). Furthermore, the presence of a potentially damaging variant in the *JAK3* kinase may influence the cytokine receptor-mediated intracellular signal transduction, with crucial implications in the differentiation, function and survival of B and T cells (Figure 3B) (54). Other immune pathways that may be influenced by this group of variants are: Fcγ R-mediated phagocytosis (*CD45*), TNF signaling-pathway (*ICAM1, JUN*), Toll-like receptor signaling-pathway (*TLR1, JUN*) and innate immune responses (*ICAM1, TLR1, MBL2*) (55). *TLR1* encodes for a member of the Toll-like receptor family, and plays a fundamental role in pathogen recognition and activation of innate immunity (30, 56). *TLR1* was identified as a critical innate receptor for protective intestinal Th17 immunity (57) but, to our knowledge, has not been previously associated with increased susceptibility to infections, or hypogammaglobulinemia.

Two potentially damaging variants were identified in *ICAM1*, which encodes a cell surface glycoprotein involved in cell-cell adhesion, expressed on endothelial cells and cells of the immune system, and has a prominent role in several types of immune responses (51, 52). Variants in *ICAM1* have been associated with inflammatory bowel disease (58). The p.G241R variant, which the twins inherited from their father, has been associated with adult onset celiac disease in a French cohort (59).

Flow-cytometry is crucial to the functional validation of genetic variants, allowing adequate interpretation of NGS data and stratification of patients (25, 26, 60). Notably, we documented a synchronous progressive B-cell depletion throughout follow-up. Taking advantage of the EuroFlow strategy for highly-sensitive Ig-subclass analysis of blood B cells and plasma cells, six subgroups of patients were recently identified in CVID, with different IgG-switching patterns and clinical profiles, even in patients with <1% B cells (7). The immunological phenotype of both twins at 50, was compatible with the CVID-6 subgroup, the most severe, defined by markedly decreased CD27^+^ unswitched and switched-memory B cells, with very low CD27^−^IgG3^+^ memory B cells. CVID-6 patients also show significantly reduced pre-germinal B cells, reflecting defective B-cell production in the bone marrow (61, 62). These recently developed standardized flow-cytometry assays to analyse memory B-cell immunoglobulin isotypes and IgH-subclasses will be very important in longitudinal studies to investigate the progression of B-cell defects, that has been hypothesized, but not yet supported by solid immunological data (7, 8).

The remarkable similarity between the immune profiles of the MZ twins, at age 50, even though they have lived in different households since they were 25, with distinct jobs and nuclear families, is even more striking in light of studies that have emphasized the dominant contribution of non-heritable influences to the shape and function of the immune system (1, 2). Although they live in the same geographical region, the impact of co-habitation has been considered very relevant in the shaping of the immune system, as illustrated by the immunological data from non-related housemates (63, 64). This debate on relative contributions of “nature” vs. “nurture” was addressed in a study that included 105 pairs of healthy MZ twins, which shows that variation in immune cell frequencies and serum proteins between twins increases with age, likely due in large part to exposure to pathogens, namely CMV (2). Notably, there has been no evidence of CMV infection in the MZ twins, both with negative PCR for CMV in blood on different occasions, and no evidence of CMV infection in gut biopsies.

Epigenetic modifications necessarily contribute to the discordance in clinical and immunological phenotype between MZ twins (1, 3). In line with this, a pair of MZ twins discordant for CVID was previously reported (3), with a significant increase in DNA methylation of B cells in the affected sister (3, 65). Consistent with this finding, a recent study showed that impaired demethylation in B-cell key genes is associated with the reduction of memory B cells in CVID patients (66). In our context, it will be interesting to explore the epigenetic landscape of the MZ concordant twins in order to confirm its contribution to the disease and add a new layer of insight to our clinical, immunological, and genomic data.

## Concluding Remarks

The clinical, immunologic and genetic profile of a pair of monozygotic twins concordant for CVID provides further support to the hypothesis that a combination of allelic variants can additively predispose to non-familial CVID. Moreover, these data suggest that genetic variants may impose a significant constraint on the impact of the environment, as attested by the remarkably similar immunological phenotype observed in both twins, despite prolonged exposure to different living conditions. The integration of NGS data with clinical and immunological phenotypes at the single-cell level, as provided by multi-dimensional flow-cytometry, is crucial to further expose the complex genetic landscape underlying the vast majority of patients with CVID, and patients with other immunodeficiencies.

## Data Availability Statement

All datasets generated for this study are included in the manuscript/supplementary files.

## Ethics Statement

The studies involving human participants were reviewed and approved by Ethical boards of the Faculty of Medicine of University of Lisbon and Centro Hospitalar Universitário Lisboa Norte. The patients/participants provided their written informed consent to participate in this study. Written informed consent was obtained from the individuals for the publication of any potentially identifiable images or data included in this article.

## Author Contributions

SLS, MF, ABS, RV, and AES designed the study. SLS, MF, SPS, and RV collected the clinical data. MF, RB, AS-C, EB, and MP-A performed the immunological studies. MF, MP, PR, AR, MG-C, and LH analyzed WES data and investigated the selected variants. SLS, MF, ABS, AR, MG-C, LH, and AES discussed the results. SLS and AES supervised the study. SLS wrote the paper.

### Conflict of Interest

The authors declare that the research was conducted in the absence of any commercial or financial relationships that could be construed as a potential conflict of interest.
